# Assessing the reliability and validity of the ICECAP-A instrument in Chinese type 2 diabetes patients

**DOI:** 10.1186/s12955-020-01632-5

**Published:** 2021-01-06

**Authors:** Yao Xiong, Hongyan Wu, Judy Xu

**Affiliations:** 1 School of Public Administration, Southwestern University of Finance and Economics, Chengdu, 611130 China; 2 Center of Health Policy and Governance, Southwestern University of Finance and Economics, Chengdu, 611130 China; 3 School of Medicine and Health Management, Guizhou Medical University, Guiyang, 550025 China

**Keywords:** ICECAP-A, EQ-5D-3L, Psychometrics, China, Diabetes

## Abstract

**Purpose:**

We aimed to conduct psychometric tests for the Chinese version of ICECAP-A and compare the differences between ICECAP-A and EQ-5D-3L for patients with T2DM and explore the relationship between clinical conditions and ICECAP-A through diabetes-related clinical indicators.

**Methods:**

Data were collected from a sample of 492 Chinese T2DM patients. The reliability and validity of the ICECAP-A were verified. Exploratory factor analysis (EFA), correlation analysis and regression analysis were conducted for both the ICECAP-A and EQ-5D-3L.

**Results:**

Our results show that the Chinese version of ICECAP-A has good internal consistency with an overall Cronbach’s Alpha coefficient of 0.721. The mean scores of ICECAP-A and EQ-5D-3L are 0.85 vs. 0.94. A weak correlation (r = 0.116) was found between the ICECAP-A tariff and EQ-5D-3L utility. EFA showed that although the five dimensions of the ICECAP-A and EQ-5D-3L scales were loaded into two different factors respectively. However, the two scales captured different dimensions of quality of life and can complement each other. The ICECAP-A, EQ-5D-3L, and EQ-VAS scores showed differences across different socio-demographic characteristics and clinic conditions groups.

**Conclusion:**

The Chinese version of the ICECAP-A capability instrument can be for assessing outcomes in adults with T2DM. It may capture more dimensions of QoL than traditional Health-related QoL (HRQoL) instruments and may be useful for economic evaluations of health care and social care for people with T2DM or other chronic diseases.

## Key points for decision makers

The ICECAP-A can be used to access the quality-of-life aspects of T2DM for the purpose of economic evaluation.

The combined use of capability of well-being and preference-based health-related quality of life can offer a broader space for assessing quality of life for T2DM patients to decision makers.

## Background

Type 2 diabetes (T2DM) is one of the top chronic conditions in China which causes big burdens for both families and countries, with the total diabetes national prevalence in adults was 10.9%, of prediabetes, 35.7% [1]. The international diabetes federation (IDF) Diabetes Atlas 2019 estimated the total health expenditure due to diabetes was USD 109.0 billion in 2019 in China [2], and T2DM-related direct annual cost was USD 90.5 billion [3].

T2DM not only makes patients endure abnormal biochemical indicators but also has a significantly negative impact on the general quality of life (QoL) and well-being due to the disease and complications. Therefore, appropriate exercise, diet, and self-management have played important roles in diabetes management besides pharmaceutical treatment. Furthermore, the purpose of treating T2DM has become more than the control and improvement of the biochemical indicators of the patient, the more important purpose is to prevent and delay the occurrence of chronic complications of diabetes and to alleviate the adverse symptoms and distress of the disease, that is, improve their general QoL and well-being.

Health-related quality of life (HRQoL) is a composite concept of a person’s subjective evaluation of their health status, which could be measured by EuroQol-5 dimension (EQ-5D), Short Form-6 dimension (SF-6D), etc. Currently, EQ-5D is the most commonly used HRQoL measurement instrument in China, including T2DM and other 17 chronic non-communicable diseases (CNCDs) [4]. As a universal preference-based HRQoL scale, EQ-5D has been widely used to measure the effects of T2DM, and EQ-5D utility associated with T2DM and various comorbidities can be very useful in the economic assessment of model health status in T2DM patient health programs [5]. However, the impacts of disease and the effects of interventions on T2DM were not limited to the HRQoL but also encompassed broader QoL and well-being [6]. The HRQoL instrument may undervalue the outcomes of the integrated care of diabetes management and the wholly negative impact of T2DM.

The capability approach is an appropriate framework for conceptualizing these broader feelings of QoL and well-being into health decisions [7, 8]. In recent years, a family of generic instruments called Investigating Choice Experiences for the Preferences of Older People (ICEpop), that has developed to measure capability (CAP) to measure more general well-being than the traditional framework of HRQoL permits [7, 9]. Unlike the popular HRQoL instruments of EQ-5D, ICECAP is designed to measure people’s function and capability based on Sen’s capability theory [10].

With a growing interest in using ICECAP instruments and the capability of a broader range of population groups, the ICECAP-A capability index has been developed to measure the generic QoL for the adult population. The ICECAP-A includes five dimensions of Stability (feel settled and secure), Attachment (have love, friendship and support), Autonomy (be independent), Achievement (achieve and progress), and Enjoyment (have enjoyment and pleasure) [11]. Unlike most profile measures used in economic evaluations, the ICECAP-A focuses on wellbeing defined in a broader sense, rather than health. In addition to studies in the general population, which also involved the comparison of ICECAP-A tariff scores for diseases with healthy populations, such as knee pain [12], depression [6], and patients with other chronic conditions. It has the potential for economic evaluations of public health interventions as well as other social care in addition to clinical-focused medical services and pharmaceutical products.

ICECAP-A has been translated into Chinese with the adaptation of Chinese culture, and the study also shows that the ICECAP-A tariff reflected differences across different socioeconomic groups as expected [9]. However, it is not clear whether ICECAP-A can distinguish subpopulations with different health conditions (e.g., T2DM) in Chinese culture since it doesn’t include respondents’ objective health status. The aims of this study are 1) Conduct psychometric tests for the Chinese version of ICECAP-A and compare the differences between ICECAP-A and EQ-5D-3L for patients with T2DM; 2) Explore the relationship between clinical conditions and ICECAP-A through diabetes-related clinical indicators.

## Methods

### Research design and setting

The data is from the Community Diabetes Management Study (CDMS), 1 which is conducted by our research team. Considering the geographical location, level of economic and social development, and accessibility, we respectively selected two community health service centers in Beijing and Chengdu. We retrieved health records from the local Center for Disease Control and Prevention and included all individuals with a previous diagnosis of T2DM living in four districts as the sampling frame. CDMS is a longitudinal panel study from June 2015 to December 2017, that includes a sample of 967 diabetes patients and 20 physicians who conduct disease management for them. The baseline survey was conducted in June 2015, the six follow-up surveys were conducted in September and December 2015, June and December 2016, and June and December 2017, respectively. That is the study had been conducted every 3 months in the first year and every 6 months in the second and later years, with a total of 7 wave surveys available now.

The participant inclusion criteria were: 1) aged 18 years or older; 2) clinically diagnosed with type 2 diabetes; 3) without any cognitive impairment and serious vision and hearing problems; 4) able to read and communicate in Mandarin; and 5) consent to participate in the study. Informed consent was obtained from all patients included in the study.

The observation exclusion criteria were: 1) loss to follow-up; 2) missing most data in socio-demographic characteristics; 3) illogical data (i.e. age was less than the duration of diabetes); 4) HbA1c = 0% or HbA1c > 20% (195 mmol/mol).

### Data collection procedure and data sources

A total of 967 patients were contacted during the survey, of whom 110 refused to be interviewed or were lost, and a further 365 were excluded from the sample based on data inclusion and screening criteria and missing values for key variables. Patients were invited to the community health service centers for face-to-face paper-and-pencil interviews at baseline and every wave follow-up. At every interview, patients received a medical examination including blood pressure. A fasting blood sample was collected to test the blood lipids, HbA1c level, and fasting blood glucose level. Each participant was also asked to complete a long-form questionnaire, which consisted of 1) socio-demographic characteristics such as age, gender, marital status, monthly income, education level, work status, and health insurance; 2) personal health information, including self-reported happiness, self-reported health status, other chronic diseases, and comorbidities; 3) QoL, which was measured by both the Chinese versions of ICECAP-A (ICECAP-A was firstly added in the 4th wave survey) and EQ-5D-3L.

The study adhered to the Declaration of Helsinki and ethics approval was obtained from the Institutional Review Board of the Fu Xing Hospital, Capital Medical University (Approval Number: 201FXHEC-KY). Written informed consent was obtained from each participant at the recruitment stage of the study.

The interviewers attended a one-day training session which included an introduction of the study, explanations for possible questions, and mock interviews. Throughout the data collection process, every filled questionnaire was checked by two other interviewers independently. A double-entry method was adopted to ensure the accuracy of data entry.

### Instruments

The ICECAP-A was developed as five dimensions (Stability, Attachment, Autonomy, Achievement, and Enjoyment), and each dimension contains four levels (ranging from no capability to full capability). In the study, the overall ICECAP-A tariff was calculated using the UK value set [13], can be transformed into index scores that range from 0 to 1, higher score means more capability.

The EQ-5D-3L consists of five dimensions with three-level options and EQ VAS (visual analogue scale). The five dimensions include Pain/Discomfort, Self-care, Usual Activities, Mobility and Anxiety/Depression. Scores for the five dimensions, by applying EQ-5D-3L value sets. We used the EQ-5D-3L utility values set that was developed by Liu GG et al., based on the Chinese urban population [14], can be transformed into index scores that range from − 0.149 to 1, higher score means better QoL.

Individual attributes for the ICECAP-A questionnaire ranged from the full capability level (4) to the no capability level (1), whereas each dimension of the EQ-5D-3L questionnaire ranges from the highest level (1) to the lowest (3). Therefore, the full capability status is (4,4,4,4,4) whilst the highest EQ-5D-3L status is (1,1,1,1,1). Because ICECAP-A and EQ-5D-3L did not exhibit the same ordinal direction for different dimensional levels, we transformed the EQ-5D-3L level values to facilitate comparison between the two scales, as a result, level (1) was reassigned as level (3) and level (3) was reassigned as level (1) in EQ-5D-3L, and the highest EQ-5D-3L status was (3,3,3,3,3).

### Analysis

The socio-demographic and clinic conditions characteristics and QoL of T2DM patients are summarized using descriptive statistics means and standard deviations (SDs) for continuous variables; frequencies and percentage for categorical variables. The patients’ ICECAP-A tariff, EQ-5D-3L utility and EQ-VAS according to their characteristics are compared with each other.

Most evidence shows that ICECAP-A is reliable and valid [15–18]. A recent study conducted by our research team shows that the Chinese version of ICECAP-A also has good internal consistency and concurrent validity based on the online general population [9]. The analysis of psychometric tests for ICECAP-A in Chinese T2DM patients was conducted as shown below based on the previous study [9].

#### Reliability test

The reliability for the questionnaire as a system can be tested to check the reliability of the ICECAP-A where Cronbach’s alpha, with a value of > 0.70 is considered acceptable.

#### Validity test

Explore factor analysis (EFA) was employed to determine whether the items of the ICECAP-A and the EQ-5D-3L could be reduced to the underlying constructs. According to the guidelines [19], there were three main steps in conducting the EFA. First, two tests were used to assess the suitability of EFA, including the Kaiser-Meyer-Olkin (KMO) Measure of Sampling Adequacy and the Bartlett test of sphericity. The KMO index ranges from 0 to 1, with 0.50 considered suitable for factor analysis. The Bartlett’s Test of Sphericity should be significant (*p* < 0.05) for factor analysis to be suitable. Second, after the factors were extracted by principal components analysis (PCA), the cumulative percent of the variance and Kaiser’s criteria (eigenvalue > 1 rule) were considered for extracting the number of factors. Third, during the interpretation of the models, promax oblique rotation was applied to allow factors to be correlated [20]. Only the highest factor loading for each item was reported. To evaluate construct validity, we assessed the discriminant validity by calculating the Pearson’s correlations between each item of the ICECAP-A and EQ-5D-3L separately. We conducted a Polychoric correlation analysis between the scores for the ICECAP-A, EQ-5D-3L, and EQ-VAS to assess the convergent validity. We employed Polychoric correlation analysis instead of Pearson correlation because the former is employed when the measurement of variables was based on an ordinal scale.

Refer to the previous literatures [9, 21, 22], socio-demographic characteristics and clinic conditions were used to construct known-group analyses. The validity of identified groups was evaluated by comparing the ICECAP-A tariff and EQ-5D-3L utility for subgroup with different socio-demographic characteristics and clinic conditions using Kruskal–Wallis tests.

Refer to previous literature valuing HRQoL in T2DM [22], a multivariate ordinary least squares regression model was employed to explore the determinants of QoL. Independent variables included in the regression model were socio-demographic characteristics and clinic conditions. We used robust standard errors to the problem of heteroscedasticity and provided a more accurate measure of the true standard error of a regression coefficient [23].

The software for Windows, Stata version 15 (Stata Corp, College Station, TX, USA) is used for statistical analysis.

## Results

### Descriptive analysis

The socio-demographic characteristics and clinical condition of the participants are shown in Table 1. In total, there were 492 participants (mean age 64.02 (Sd 9.57) years) included in our study, 60.6% (*n* = 298) were female. In this study, almost 86.2% (*n* = 424) of participants had a lower than college educational level. More than 90% of the participants were married. Regarding clinical condition, 82.7% (*n* = 407) of participants had been diagnosed with other chronic diseases, and 70.3% (*n* = 344) of participants did not report any complications.

**Table 1:** Sample characteristics for the ICECAP-A and EQ-5D-3L scores and EQ-VAS by socio-demographic and clinic conditions groups of the sample

		ICECAP-A tariff			EQ-5D-3L utility			EQ-VAS		
	N	Mean	Sd	*p*-value	Mean	Sd	p-value	Mean	Sd	p-value
**Gender**				0.725			0.154			0.036
Male	194	0.85	0.09		0.95	0.06		82.98	10.59	
Female	298	0.85	0.10		0.93	0.08		80.74	10.89	
**Age**				0.005			0.595			< 0.001
< 35	2	0.94	0.05		0.96	0.00		92.50	3.54	
35–44	14	0.89	0.07		0.94	0.07		85.00	9.58	
45–54	64	0.87	0.11		0.95	0.05		85.17	7.92	
55–64	140	0.85	0.11		0.94	0.07		81.64	13.66	
≥65	272	0.84	0.08		0.93	0.08		80.51	9.59	
**Marital status**				0.017			0.007			< 0.001
Married	448	0.86	0.09		0.94	0.06		82.19	10.55	
Unmarried	44	0.81	0.12		0.90	0.11		75.80	11.88	
**Monthly income per capita**				0.197			0.694			< 0.001
< ¥1000	54	0.87	0.12		0.93	0.07		73.52	15.07	
¥1001–¥2000	57	0.86	0.09		0.92	0.12		78.75	11.87	
¥2001–¥3000	225	0.85	0.08		0.94	0.06		82.59	9.36	
¥3001–¥4000	120	0.85	0.10		0.95	0.05		83.78	8.68	
≥¥4000	36	0.83	0.11		0.93	0.11		85.03	10.29	
**Education**				0.667			0.223			< 0.001
Primary school or below	122	0.85	0.11		0.92	0.09		75.73	13.03	
Middle school	132	0.85	0.09		0.93	0.08		82.34	9.72	
High or profession school	170	0.85	0.09		0.94	0.06		83.36	9.59	
College or above	68	0.86	0.09		0.96	0.02		86.47	6.11	
**Work status**				0.021			0.756			< 0.001
Other work status	99	0.86	0.08		0.94	0.07		84.96	6.37	
Working	63	0.89	0.07		0.94	0.06		86.25	7.83	
Leisure	330	0.84	0.10		0.94	0.07		79.74	11.82	
**Health insurance**				0.004			0.645			< 0.001
Government medical insurance	3	0.87	0.01		0.96	0.00		73.33	15.28	
Urban resident medical insurance	17	0.79	0.16		0.95	0.05		81.35	6.10	
Urban employee medical insurance	359	0.85	0.08		0.94	0.07		83.83	8.93	
New cooperative medical insurance	111	0.88	0.11		0.93	0.08		74.74	13.67	
No insurance	2	0.89	0.07		0.96	0.00		85.00	0.00	
**Income source**				< 0.001			0.585			0.261
Salary	311	0.85	0.09		0.94	0.06		82.49	9.35	
Agriculture production or business	21	0.88	0.08		0.95	0.04		77.05	11.36	
Other source	127	0.83	0.10		0.93	0.10		80.40	13.31	
Unkown	33	0.92	0.10		0.93	0.06		80.91	12.08	
**Self-reported happiness**				0.012			0.022			0.001
Very happy	423	0.85	0.09		0.94	0.06		82.00	11.04	
Fairly happy	66	0.85	0.09		0.92	0.11		80.17	8.25	
fairly unhappy	3	0.55	0.08		0.81	0.05		60.00	0.00	
**Self-reported health status**				0.013			0.002			< 0.001
Good health	314	0.86	0.09		0.95	0.06		84.37	9.50	
Fair health	165	0.85	0.09		0.92	0.08		77.86	10.12	
Poor health	12	0.74	0.15		0.87	0.14		62.42	16.98	
**Number of Other Chronic diseases**				0.248			0.051			< 0.001
None	85	0.86	0.07		0.94	0.07		85.42	6.18	
1	222	0.86	0.09		0.95	0.06		83.19	9.95	
2 or above	185	0.84	0.11		0.93	0.08		78.02	12.36	
**Number of Complications**				0.631			< 0.001			< 0.001
None	344	0.85	0.09		0.95	0.06		83.42	8.66	
1	104	0.84	0.10		0.93	0.08		77.88	15.00	
2 or above	41	0.85	0.08		0.89	0.1		76.20	10.64	
**Fasting Blood Glucose**				0.184			0.401			0.001
< 6.1 mmol/L	256	0.85	0.08		0.94	0.06		83.78	7.85	
6.1 ~ 7.0 mmol/L	87	0.85	0.11		0.94	0.06		83.81	7.74	
≥7.0 mmol/L	133	0.86	0.11		0.93	0.09		77.83	14.38	
**Two hours Postprandial Blood Glucose**				0.477			0.550			0.002
< 7.8 mmol/L	88	0.84	0.10		0.94	0.05		83.76	8.75	
7.8 ~ 11.1 mmol/L	270	0.85	0.09		0.94	0.07		83.24	8.08	
≥11.1 mmol/L	88	0.86	0.10		0.93	0.09		77.18	15.26	
**HbA1c**				0.020			0.248			0.010
< 6.5%	328	0.84	0.09		0.94	0.06		83.12	8.74	
≥6.5%	141	0.87	0.09		0.93	0.09		79.21	13.54	
Overall sample		0.85	0.10		0.94	0.07		81.62	10.82	

ICECAP-A ICEpop CAPability measure for adults, EQ-5D-3L EuroQoL Three-Dimension, Sd standard deviation

There are 1, 3, 16, 42, 23 missing data which in the categories of self-reported happiness, number of complications, fasting blood glucose, two hours postprandial blood glucose, HbA1c, respectively

Monthly income per capita is classified into three levels: low-income (less than CNY 2000), middle-income (between CNY 2001 and CNY 4000), and high-income (more than CNY 4000)

The average ICECAP-A tariff in samples was 0.85. Male participants had an equal ICECAP-A tariff compared to female participants. Participants over 65 years old had the lowest ICECAP-A, EQ-5D-3L, and EQ-VAS scores compared to younger participants. Married participants and those with a higher level of education had a higher ICECAP-A, EQ-5D-3L, and EQ-VAS scores compared to those in other marital status and with lower education, respectively. Furthermore, based on the monthly income per capita, we found that high-income participants indicated the lowest ICECAP-A tariff compared with low-income participants, but middle-income participants indicated the highest EQ-5D-3L score. Concerning clinical conditions, EQ-5D-3L and EQ-VAS scores in participants without complications or comorbidities were higher than those with complications. Moreover, participants with higher HbA1c reported a higher ICECAP-A tariff and lower EQ-5D-3L and EQ-VAS scores compared to those who had lower HbA1c.

The distribution of responses to the ICECAP-A instrument is presented in Table 2. There were no more than 30% of the participants who reported full capability in the attributes of Stability and Attachment. Meanwhile, only 15.5% (*n* = 76) of the participants could make achievements and progress in all aspects of their life. On the contrary, more participants had full capability performing completely independently (52.9%) and enjoyment (36.4%).

**Table 2:** Response to ICECAP-A questionnaire

Attributes	Level 4		Level 3		Level 2		Level 1	
	n	(%)	n	(%)	n	(%)	n	(%)
**Stability**	138	28.1	334	67.9	20	4.1	0	0.0
**Attachment**	120	24.4	317	64.4	55	11.2	0	0.0
**Autonomy**	260	52.9	167	33.9	65	13.2	0	0.0
**Achievement**	76	15.5	275	55.9	139	28.3	2	0.4
**Enjoyment**	179	36.4	299	60.8	14	2.9	0	0.0

Each of dimension in ICECAP-A can take one of four levels ranging from full capability to no capability, where level 4 represents full capability and level 1 represents no capability

Different from the distribution of responses to the ICECAP-A instrument, more than 91% of the participants reported no problem in all the five attributes in the EQ-5D-3L (Table 3).

**Table 3:** Response to EQ-5D-3L questionnaire

Attributes	Level 3		Level 2		Level 1	
	n	(%)	n	(%)	n	(%)
**Mobility**	457	92.9	34	6.9	1	0.2
**Self-care**	480	97.6	12	2.4	0	0.0
**Usual activities**	471	95.7	20	4.1	1	0.2
**Pain/ discomfort**	452	91.9	39	7.9	1	0.2
**Anxiety/ depressed**	481	97.8	11	2.2	0	0.0

Each of dimension in EQ-5D-3L can take one of three levels ranging from no problem to severe problem/ unable to, where level 3 represents no problems and level 1 represents severe problem/ unable to

### Reliability test

Both the Cronbach’s Alpha coefficients are more than 0.7, which suggests an appropriate level of reliability.

### Validity test

#### Exploratory factor analysis

The factor load obtained after the promax rotation is shown in Table 4. It can be found that the five dimensions of the ICECAP scale are mainly loaded on factor 2, and the five dimensions of the EQ-5D-3L scale are mainly loaded on factor 1. The factor correlation is 0.258, meaning the promax rotation was an appropriate choice for the analysis. The results indicated that there is a different construct between the two scales, providing different meaning information in T2DM.

**Table 4:** Exploratory factor analysis comparing the ICECAP-A and EQ-5D-3L

	Rotated promax	
	Factor 1	Factor 2
**ICECAP-A**		
Stability		0.347
Attachment		0.099
Autonomy		0.208
Achievement		0.224
Enjoyment		0.279
**EQ-5D-3L**		
Mobility	0.189	
Self-care	0.225	
Usual activities	0.428	
Pain/ discomfort	0.117	
Anxiety/ depressed	0.192	
Factor correlation(s)	0.258	

Loadings of <|0.09| are dropped from the table to allow easy interpretation of results

#### Convergent and discriminative validity

Each item of the ICECAP-A and EQ-5D-3L in Chinese is independent as the correlation factor ranges from 0.063 to 0.637 (Table S1 and Table S2). The correlation between dimensions of the two scales was weak-to-moderate (polychoric correlation range − 0.335 ~ 0.561) (Table 5). Specifically, the Anxiety/depressed dimension has higher correlations with the four other dimensions except for Autonomy, where the correlation with Stability and Enjoyment exceeds 0.5. Autonomy has higher correlations with Mobility, Self-care, and Usual activities than the other ICECAP-A dimensions, where the correlation with Self-care and Usual activities exceeds 0.5. We found the weak correlations of ICECAP-A items with EQ-5D-3L utility, and EQ-VAS score (polychoric correlation range − 0.214 ~ 0.367). Weak correlations of ICECAP-A tariff were also observed with the EQ-5D-3L utility, EQ-VAS score (polychoric correlation of 0.116 and 0.218, respectively).

**Table 5:** Polychoric correlation coefficient between ICECAP-A and EQ-5D-3L

ICECAP-A	EQ-5D-3L					EQ-5D-3L utility	EQ-VAS
	Mobility	Self-care	Usual activities	Pain/ discomfort	Anxiety/ depressed		
Stability	0.266	0.454	0.243	−0.018	0.545	0.149	−0.022
Attachment	0.052	0.043	0.038	−0.335	0.109	−0.046	0.367
Autonomy	0.435	0.561	0.514	−0.128	0.093	0.172	− 0.214
Achievement	0.133	0.210	0.209	0.200	0.299	0.135	0.051
Enjoyment	0.379	0.316	0.335	−0.046	0.538	0.171	0.159
ICECAP-A tariff	0.163	0.207	0.209	−0.031	0.202	0.116	0.218

#### Identified groups’ validity

As shown in Table 1, the Kruskal Wallis test showed that the differences in the overall distribution of QoL among seven categories of the sample (e.g., age, marital status, work status, category of health insurance and level of HbA1c) were statistically significant (*P* < 0.05) for the ICECAP-A measure, and differences in the overall distribution of HRQoL among four categories of the sample (e.g., marital status, number of complications) statistically significant (P < 0.05) for the EQ-5D-3L measure.

#### Regression analysis

Several sociodemographic characteristics of the participants were shown to significantly influence the ICECAP-A tariff and EQ-VAS in the multiple regression analysis (Table 6), mostly in line with the results of the univariate analysis presented above in Table 1. The older participants had lower EQ-5D utility and EQ-VAS scores. But higher education contributed to a significantly better capability for the participants in our study. The variables about clinical conditions were almost shown to not significantly influence the ICECAP-A tariff except for self-reported happiness and self-reported health status. Not surprisingly, participants with better self-reported happiness and self-reported health status had a higher ICECAP-A tariff. The R^2^ statistics indicate that the demographics and health conditions accounted for approximately a fifth of the variance in QoL index scores (ICECAP-A = 22.4% and EQ-5D-3L = 13.7%).

**Table 6:** Ordinary least squares regression results of ICECAP-A and EQ-5D-3L on socio-demographic characteristics and clinic conditions

Variables	ICECAP-A		EQ-5D-3L		EQ-VAS	
	Coef.	S.E.	Coef.	S.E.	Coef.	S.E.
**Pannel A: Socio-demographic characteristics**						
**Gender (reference = Male)**						
Female	0.003	0.009	−0.005	0.007	1.225	0.886
**Age**	−0.001	0.001	−0.001^**^	0.000	−0.131^**^	0.051
**Marital Status (reference = Married)**						
Other marital status	0.003	0.016	−0.016	0.019	0.414	1.567
**Monthly income per capita (reference = <** ¥**1000)**						
¥1001–¥2000	0.050^**^	0.024	−0.023	0.020	7.515^**^	3.122
¥2001–¥3000	0.025	0.027	0.006	0.020	8.746^**^	3.416
¥3001–¥4000	0.015	0.028	0.003	0.020	8.837^**^	3.602
>¥4000	−0.013	0.031	−0.019	0.027	8.805^**^	3.732
**Education (referent = Primary school or below)**						
Middle School	0.032^**^	0.016	0.009	0.015	1.798	1.491
High or profession school	0.035^**^	0.016	0.009	0.016	1.349	1.629
College and above	0.052^**^	0.019	0.021	0.018	3.660^**^	1.692
**Working status (reference = Working)**						
Leisure	−0.002	0.015	−0.02*	0.012	−0.273	1.288
Other status	−0.035^**^	0.012	0.002	0.010	−1.720	1.096
**Health insurance (referent = No insurance)**						
Government medical insurance	−0.025	0.055	−0.028	0.049	−1.982	4.821
Urban employee medical insurance	−0.029	0.032	−0.031	0.035	2.576	2.056
Urban resident medical insurance	−0.091	0.050	−0.014	0.035	−0.567	2.570
New cooperative medical insurance	0.024	0.039	−0.012	0.041	−1.985	3.506
**Income source (referent = Salary)**						
Agriculture production or business	−0.069^**^	0.026	−0.012	0.019	−12.583^**^	3.597
Other income source	−0.113^**^	0.029	−0.002	0.023	−12.489^**^	3.717
Unknown	−0.113^**^	0.025	−0.027	0.018	−10.919^**^	3.445
**Pannel B: Clinic conditions**						
**Self-reported happiness(referent = Fairly unhappy)**						
Fairly happy	0.404^**^	0.034	0.071**	0.036	21.296^**^	3.163
Very happy	0.400^**^	0.033	0.076^**^	0.032	20.796^**^	3.100
**Self-reported health status(referent = Poor health)**						
Fair health	0.086^**^	0.044	0.007	0.049	8.977^**^	5.032
Good health	0.093^**^	0.044	0.024	0.048	13.261^**^	4.963
**Number of other chronic diseases (reference = No** **other chronic diseases)**						
One	0.011	0.011	0.014	0.010	−0.875	0.916
Two or above	−0.013	0.013	−0.001	0.009	−2.366^*^	1.112
**Number of complications (reference = No complication)**						
One	−0.006	0.012	0.004	0.008	−1.851^*^	1.797
Two or above	0.017	0.019	−0.022	0.018	−2.375	1.920
**FPG (referent = 6.1–7.0 mmol/L)**						
< 6.1 mmol/L	−0.002	0.016	−0.005	0.012	−4.403^**^	1.442
6.1–7.0 mmol/L	0.003	0.013	0.005	0.007	−0.787	0.953
**PG2H (referent = < 7.8 mmol/L)**						
7.8 ~ 11.1 mmol/L	−0.002	0.013	−0.008	0.012	−2.349	1.708
≥ 11.1 mmol/L	−0.010	0.011	−0.003	0.007	0.162	0.918
**HbA1c (referent = < 6.5%)**						
≥6.5%	0.018*	0.011	−0.006	0.011	1.001	1.117
***R***^**2**^	0.224		0.137		0.367	
***N***	426		426		423	

Coef. coefficient, S.E. standard error

*significant at the 10% level; **significant at the 5% level

## Discussion

The Cronbach’s alpha coefficient was 0.72 for the ICECAP-A in this study, which was lower than the previous results in the Chinese general population (0.80) [9], German T2DM patients (0.83), and English T2DM patients (0.86) [17]. However, a value of Cronbach’s alpha coefficient > 0.7 is acceptable to test the internal consistency of an instrument.

Overall, the dimensions of the two scales were weakly to moderately correlated (0.2~0.6), which is consistent with the general population [9, 18] and women with irritative lower urinary tract symptoms [16]. However, in significant contrast to these studies, we also observed weak negative correlations among ICECAP-A, EQ-5D-3L, and EQ-VAS. The possible reasons are as follows: Among the five dimensions of EQ-5D-3L, Mobility, Self-care, Physical activities, and Pain/discomfort are more related to physical health, and among these dimensions, the Chinese population perceives that the three dimensions of Mobility, Self-care, and Physical activities had a significantly greater impact on health-related quality of life than the Pain/discomfort dimension [24], the problems appearing in the dimensions of Mobility, Self-care, and Usual activities will not only affect patient’s QoL, but will also significantly affect that family members engage in a multitude of essential activities for patients. Whereas more than 80% of the respondents in this study were over 55 years old and 55% were older than 65 years, in China, most older people live with their children, and when they experience mild discomfort such as Pain/discomfort, their families and children can give them more emotional and psychological family supports, therefore, these respondents may experience higher capability on the dimensions of Stability, Attachment, Autonomy, and Enjoyment. It was also observed in the study that the Pain/discomfort dimension was positively correlated with the Achievement dimension, which was closely related to respondents’ capability. The correlation coefficients of ICECAP-A with EQ-5D-3L and EQ-VAS were lower than those in knee pain [12] and irritative lower urinary tract symptoms [16], but consistent with these studies, ICECAP-A was weakly correlated with EQ-5D-3L than with EQ-VAS [25].

In terms of discriminative validity, there were statistically significant associations between measured capability and age, marital status, work status, insurance, income source, and HbA1c, while there were only significant associations between measured HRQoL and marital status and number of complications, but both the two instruments were significant associations with self-reported health status and self-reported happiness. However, based on the results in regression, the independent variables with statistically significant coefficients in ICECAP-A were similar to EQ-VAS.

In terms of construct validity, although the Anxiety/depressed dimension had the highest correlation with the four dimensions in the ICECAP-A, the Anxiety/depressed dimension was still loaded with factor 1 with the other four dimensions of the EQ-5D-3L in the factor analysis, which was different with the general population [9, 18] and patients with knee pain [12], where the Anxiety/depressed dimension was loaded with another factor with the five dimensions of the ICECAP-A.

Across the five dimensions in ICECAP-A, the T2DM patients had the least number of people at the full capability level in the Achievement dimension and the most number of people at the full capability level in the Autonomy dimension, consistent with the results of the Chinese general population [9]. However, although elder participants with disease, this study showed that means values of the Chinese T2DM patients for the ICECAP-A, EQ-5D-3L, and EQ-VAS were higher than those in the Chinese general population [9]. The higher values of our study might be explained by the population’s adaptation to disease and age [17, 26–29]. In terms of the effect of disease, the lower score might be due to disease, but on the other hand, the score may not be significantly lower or higher due to the population’s adaptation to disease; in terms of the effect of age, some studies show a positive or negative effect on the score with increasing age, with the reasons that the increased impact of disease with age, especially in the elderly population, and the increased adaptability of the population with age [17, 30].

This study has a few limitations that are worthy of discussion:

First, in the Community Diabetes Management Study (CDMS), ICECAP-A was added in the 4th to the last wave survey (six months interval was set in the adjacent surveys) to evaluate the impact of health management conducted by physicians on Chinese T2DM patients. Therefore, the data is inadequate to conduct the test-retest reliability analysis because of the long follow-up time interval and the interventions carried out in the study. In addition, adaptation and assessments of the Chinese version of the ICECAP-A was first conducted in 2016 [9]; however, the test-retest reliability analysis was also lacked in this study because of a one-time online survey. Therefore, to evaluate the test-retest reliability of the ICECAP-A will be an important topic of research [31, 32].

Second, the ICECAP-A instrument was not included in the previous waves’ questionnaires, further investigation can be undertaken to use longitudinal data to test the correlation of ICECAP-A changes and diabetes clinical outcome changes.

Third, the sample size for this study is small and concentrated in patients who are older than 65-years old. Thus, the conclusion should be cautious when it is promoted and applied to T2DM patients nationwide. Further studies with other chronic patients and compared to the ICECAP-O instrument are needed to add evidence to the international literature on the validity and use of the ICECAP-A.

Fourth, the ICECAP-A tariff is based on the UK value set, and studies have shown that the numerical differences in the EQ-5D scores obtained from the conversion tables of different countries are statistically significant [33–35]. For this reason, it is necessary to develop a Chinese ICECAP-A value set to conduct economic evaluation.

Finally, a self-reported health survey may suffer reporting heterogeneity [36], different populations have different understandings of the meaning of the same concept, e.g., women consider more emotional aspects of health than men when making self-assessment of overall health [37]; even if there is a consistent understanding of the meaning of the measured health concept, different groups may have different judgments about the actual level represented by a uniform response option [38]. This was not explored in the current work but could however be investigated by using anchoring vignette method to examine the effects of response heterogeneity in the self-reported capability survey.

In this study, when the ICECAP-A scale was validated in the T2DM population, there were differences in the correlation with the EQ-5D-3L scale and factor loading with the general population and other disease populations and may be partly due to heterogeneity of disease or population. Therefore, studies on the measured properties of ICECAP-A in other disease populations could be conducted in the future.

## Conclusion

This study was conducted to assess the reliability and validity of ICECAP-A in Chinese T2DM patients and explore the correlations of the ICECAP-A with the EQ-5D-3L. Despite cross-cultural differences between countries, our study suggested that the Chinese version of the ICECAP-A was able to measure QoL of T2DM in China. The results provided supporting evidence for the reliability and validity of the adapted version of the ICECAP-A.

This is the first paper to provide evidence that the use of a capability instrument in Chinese T2DM patients and aims to explore the ICECAP-A for measuring border well-being and QoL. Although despite some limitations, the results demonstrate the appropriateness of ICECAP-A for the reflection of diabetes capability.

## Supplementary Information

**Additional file 1. Table S1**: Correlation between each item in ICECAP-A. **Table S2**: Correlation between each item in EQ-5D-3L.

## Data Availability

The datasets generated during and/or analysed during the current study are available from the corresponding author on reasonable request.

## Footnotes

1 CDMS is conducted by the Center of Health Policy and Governance (CHPG) at Southwestern University of Finance and Economics (SWUFE) with funding support from National Nature Science foundation of China (NSFC) and Sichuan Education Department Fund for Innovative Teams. The protocol was approved by the institutional review board and all participants.
