# Supplementary material for: Assessing the reliability and validity of the ICECAP-A instrument in Chinese type 2 diabetes patients
Source: Health Qual Life Outcomes. 2021 Jan 6;19:5. doi: 10.1186/s12955-020-01632-5 (PMC7788876; doi:10.1186/s12955-020-01632-5)
Supplement: Supplementary file 1 — Additional file 1. Table S1: Correlation between each item in ICECAP-A. Table S2: Correlation between each item in EQ-5D-3L. [file 12955_2020_1632_MOESM1_ESM.docx]

**Title Page**

**Journal name:**

***Health and Quality of Life Outcomes***

**Title:**

Assessing the reliability and validity of the ICECAP-A instrument in Chinese Type 2 Diabetes Patients

**Authors:**

**Yao Xiong^1,2,†^, Hongyan Wu^3,†,*^, Judy Xu^1,2^**

**Affiliations:**

^1^ School of Public Administration, Southwestern University of Finance and Economics, Chengdu, 611130, China;

^2^ Center of Health Policy and Governance, Southwestern University of Finance and Economics, Chengdu, 611130, China

^3^ School of Medicine and Health Management, Guizhou Medical University, Guiyang, 550025, China;

*****Correspondence: why_vivian@163.com

†Equal contributors

**Abstract**

**Purpose**  We aimed to conduct psychometric tests for the Chinese version of ICECAP-A and compare the differences between ICECAP-A and EQ-5D-3L for patients with T2DM and explore the relationship between clinical conditions and ICECAP-A through diabetes-related clinical indicators.

**Methods** Data were collected from a sample of 492 Chinese T2DM patients. The reliability and validity of the ICECAP-A were verified. Exploratory factor analysis (EFA), correlation analysis and regression analysis were conducted for both the ICECAP-A and EQ-5D-3L.

**Results** Our results show that the Chinese version of ICECAP-A has good internal consistency with an overall Cronbach's Alpha coefficient of 0.721. The mean scores of ICECAP-A and EQ-5D-3L are 0.85 vs. 0.94. A weak correlation (r = 0.116) was found between the ICECAP-A tariff and EQ-5D-3L utility. EFA showed that although the five dimensions of the ICECAP-A and EQ-5D-3L scales were loaded into two different factors respectively. However, the two scales captured different dimensions of quality of life and can complement each other. The ICECAP-A, EQ-5D-3L, and EQ-VAS scores showed differences across different socio-demographic characteristics and clinic conditions groups.

**Conclusion** The Chinese version of the ICECAP-A capability instrument can be for assessing outcomes in adults with T2DM. It may capture more dimensions of QoL than traditional Health-related QoL (HRQoL) instruments and may be useful for economic evaluations of health care and social care for people with T2DM or other chronic diseases.

**Keywords** ICECAP-A EQ-5D-3L Psychometrics China Diabetes

**Key points for decision makers**

The ICECAP-A can be used to access the quality-of-life aspects of T2DM for the purpose of economic evaluation.

The combined use of capability of well-being and preference-based health-related quality of life can offer a broader space for assessing quality of life for T2DM patients to decision makers.

**Declarations**

**Funding** This research was supported by the National Natural Science Foundation of China (Grant No. 71463007 & 71704144).

**Conflict of interest** All authors declare that they have no conflict of interest.

**Availability of data** **and material** The datasets generated during and/or analysed during the current study are available from the corresponding author on reasonable request.

**Ethics approval** All procedures performed in studies involving human participants were following the ethics approval, which was obtained from the Institutional Review Board of the Fu Xing Hospital, Capital Medical University (Approval No. 201FXHEC-KY), and with the 1964 Helsinki declaration and its later amendments or comparable ethical standards.

**Consent** Informed consent was obtained from all individual participants included in the study.

**Author contributions** Judy Xu and Hongyan Wu developed the idea. All authors contributed to the study conception and design. Data collection and analysis were performed Judy Xu and Yao Xiong. The manuscript was written by Yao Xiong and Hongyan Wu and all authors commented on previous versions of the manuscript. All authors read and approved the final manuscript.

**Acknowledgments**

We are particularly grateful for the valuable suggestions from Professor Joanna Coast at the University of Bristol and Professor Shunping Li at the Shandong University for our research work. The opinions expressed in this article and any errors are those of authors alone.

Table S1. Correlation between each item in ICECAP-A

| **Attributes** | Stability | Attachment | Autonomy | Achievement | Enjoyment |
| --- | --- | --- | --- | --- | --- |
| Stability | 1.000 |  |  |  |  |
| Attachment | 0.207* | 1.000 |  |  |  |
| Autonomy | 0.448* | 0.178* | 1.000 |  |  |
| Achievement | 0.559* | 0.063 | 0.402* | 1.000 |  |
| Enjoyment | 0.535* | 0.351* | 0.404* | 0.393* | 1.000 |
| Cronbach's Alpha | 0.721 | | | | |

Note: *All coefficients are significant at the 1 % level

Table S2. Correlation between each item in EQ-5D-3L

| **Attributes** | Mobility | Self-care | Usual activities | Pain/ discomfort | Anxiety/ depressed |
| --- | --- | --- | --- | --- | --- |
| Mobility | 1.000 |  |  |  |  |
| Self-care | 0.448* | 1.000 |  |  |  |
| Usual activities | 0.610* | 0.637* | 1.000 |  |  |
| Pain/ discomfort | 0.214* | 0.186* | 0.271* | 1.000 |  |
| Anxiety/ depressed | 0.318* | 0.422* | 0.477* | 0.392* | 1.000 |
| Cronbach's Alpha | 0.727 | | | | |

Note: *All coefficients are significant at the 1 % level
